# Memories without Survival: Personal Identity and the Ascending Reticular Activating System

**DOI:** 10.1093/jmp/jhad028

**Published:** 2023-06-14

**Authors:** Lukas J Meier

**Affiliations:** University of Cambridge, Cambridge, United Kingdom

**Keywords:** ascending reticular activating system (ARAS), consciousness, John Locke, memory, mental states, personal identity

## Abstract

Lockean views of personal identity maintain that we are essentially persons who persist diachronically by virtue of being psychologically continuous with our former selves. In this article, I present a novel objection to this variant of psychological accounts, which is based on neurophysiological characteristics of the brain. While the mental states that constitute said psychological continuity reside in the cerebral hemispheres, so that for the former to persist only the upper brain must remain intact, being conscious additionally requires that a structure originating in the brainstem—the ascending reticular activating system—be functional. Hence, there can be situations in which even small brainstem lesions render individuals irreversibly comatose and thus forever preclude access to their mental states, while the neural correlates of the states themselves are retained. In these situations, Lockeans are forced to regard as fulfilled their criterion of diachronic persistence since psychological continuity, as they construe it, is not disrupted. Deeming an entity that is never again going to have any mental experiences to be a person, however, is an untenable position for a psychological account to adopt. In their current form, Lockean views of personal identity are therefore incompatible with human neurophysiology.

## I. INTRODUCTION

Ever since the publication of John Locke’s *Essay Concerning Human Understanding* in the late seventeenth century, views that focus on psychological relations have been dominating the debate on personal identity. Many of those who advocate psychological accounts believe, as Locke did, that we persist over time by virtue of being linked to our former selves through a continuity in mental states. However, certain medical conditions can destroy some of these states while the patient remains conscious and able to communicate, and it was pointed out that such conditions pose a threat to Lockean views of personal identity:individuals who suffer from Alzheimer’s disease or from retrograde amnesia may be unable to recall past events, plans that they had made, or beliefs that they had held; yet it would be absurd to assume that these subjects have ceased to exist, as Lockean accounts imply (Gervais, 1986, 117–118; McMahan,1995, 110–111, 2002, 43–44).

In this paper, I argue that there also exists an opposite permutation that is equally threatening to Lockean accounts of personal identity: a configuration in which an individual’s mental states persist although his or her brain’s capacity to support consciousness is irreversibly lost. This pattern occurs when the ascending reticular activating system (ARAS), a neural network that originates in the brainstem, is rendered dysfunctional, while the rest of the brain remains intact.

I begin by briefly recapitulating the criterion of diachronic personal identity that Lockeans endorse. I then explain the role that the ARAS plays in the generation of consciousness before examining how damage to this structure engenders a situation that is incompatible with Lockean accounts as they are currently formulated. I also consider three possible objections to my argument: that a person’s mental states persist only when the ARAS remains functional, that a dysfunctional ARAS exerts a qualitative influence on mental states, and that the ARAS is a replaceable structure. I conclude by suggesting how Lockean views could be modified to account for the neurophysiological characteristics of the human brain and thereby be immunized against the challenge presented in this paper.

## II. THE LOCKEAN CRITERION OF DIACHRONIC PERSONAL IDENTITY

Famously, Locke put forward the following criterion for the diachronic persistence of persons:

Whatever has the consciousness^1^ of present and past Actions, is the same Person to whom they both belong. Had I the same consciousness, that I saw the Ark and *Noah*’s Flood, as that I saw an overflowing of the *Thames* last Winter, or as I write now, I could no more doubt that I, that write this now, that saw the *Thames* overflow’d last Winter, and that view’d the Flood at the general Deluge, was the same *self*. (2008, II.XXVII, §16)

According to this definition, an individual *y* at *t*_2_ is identical with an individual *x* at *t*_1_ if and only if *y* can remember having had *x*’s experiences. Hence, direct memory connections are both necessary and sufficient for a person to continue to exist. This condition, however, leads to an inconsistency: if an individual *x* at *t*_1_ is psychologically connected to an individual *y* at *t*_2_, and *y* is psychologically connected to an individual *z* at *t*_3_, it does not follow that *x* is psychologically connected to *z*. This cannot be right, however, since when their respective psychological connections link both *x* and *z* to the same biographical event at *t*_2_, they are supposed to be stages of the same person (Reid, 1878, III.VI, § 15–30). It was therefore suggested instead to rely on a *continuity* of memory. According to this broader criterion, individual *x* at *t*_1_ is identical with individual *y* at *t*_2_ if and only if there is an overlapping chain of memories between the two points in time (Shoemaker and Swinburne, 1984, 90; Parfit, 1987, 205).

Modern proponents of Lockean views, so-called *neo-Lockeans*, have modified Locke’s original account even further.^2^ Memories understood as recollections of autobiographical events constitute merely one of many different kinds of mental states. Other types of states include desires, intentions, perceptions, values, attitudes, beliefs, and personality traits. Nowadays, most Lockeans therefore hold that not only a continuity of memories that depict autobiographical events but also the persistence of all these other types of mental states accounts for a person’s diachronic existence (Nagel, 2013, 276; Shoemaker, 2008, 316; 1999, 288; Noonan, 2003, 10–11; Wollheim, 1999, 2; Parfit, 1987, 205; Nagel, 1986, 45).

We can now specify what neo-Lockeans put forward as the criterion of transtemporal personal identity: an individual *x* at *t*_1_ is psychologically continuous, and hence, identical, with an individual *y* at *t*_2_ if and only if there is a sufficient number of psychological connections that need not extend from *t*_1_ to *t*_2_, but that form overlapping chains between these two points in time (Shoemaker and Swinburne, 1984, 90; Parfit, 1987, 206–207).^3^ A psychological connection is established by individual *x* at *t*_1_ and individual *y* at *t*_2_ both possessing the same mental state *a*. This mental state may be an autobiographical memory or any other type of state. For the sake of simplicity, I henceforth collapse the distinction between psychological continuity and connectedness.

## III. THE ASCENDING RETICULAR ACTIVATING SYSTEM AND THE PERSISTENCE OF PERSONS

Many authors have provided definitions of consciousness, but to date none of them can count as being universally accepted (Damasio, 2010, 157–159; McGinn, 2008, 237–238; Zeman, 2002, 13–16; Block, 1995, 227–228; Nagel, 1974, 436). In this article, I employ the definition used in clinical practice. It distinguishes two major aspects of consciousness that are easily conflated: its quantitative and its qualitative dimension. One may denote the former as *wakefulness* and the latter as *awareness* (Multi-Society Task Force on PVS, 1994, 1501).

Unsurprisingly, the basis for someone to be conscious is for him or her to be awake—as opposed to being asleep or comatose. Under normal circumstances, wakefulness—which is also denoted as *arousal* or *vigilance*—is a daily recurring brain state. Wakefulness can be conceived of as the *level* of consciousness, encompassing many intermediate stages: a subject may be somnolent, fully alert, or anything in between; just as one may be lightly or deeply anesthetized (Zeman, 2006, 358). Hence, wakefulness is a graded notion. As we shall see, the regulation of the level of wakefulness is predominantly a function of areas in the brainstem from which the ARAS originates.

When one is awake, however, one is usually also aware *of* something; the stream of consciousness has a certain *content*, like a perception of an internal or external stimulus that one is having, an emotion that one is feeling, or a memory that one is recalling. Being aware therefore means that, in addition to having reached a sufficient level of wakefulness, certain processes are taking place that give rise to a stream of consciousness. Awareness is predominantly a function of complex interactions between several areas in the cerebrum (Plum and Posner, 1980, 11; Pallis and Harley, 1996, 10).

Together, wakefulness and awareness constitute consciousness. The relation between these two components is a hierarchical one. Although wakefulness does not predispose the content of the stream of consciousness, that is, it does not determine or influence *of what* the subject is aware, being awake is a necessary condition for awareness to manifest. The converse is not the case: wakefulness can occur without awareness, and when it does, the result is the so-called *vegetative state* (Meier, 2020). Patients in this condition open their eyes and exhibit sleep–wake cycles, but profound damage to the cerebral hemispheres precludes this arousal from being associated with any experiential content (Multi-Society Task Force on PVS, 1994, 1500–1501; Giacino et al., 2018, 2). The vegetative state may therefore be described as *wakeful unawareness*.

The quest for the mechanism that underlies the regulation of wakefulness and sleep began in the last quarter of the nineteenth century. Initially, it was thought that sleep is a passive phenomenon that results from a lack of adequate sensory stimulation, which was considered necessary to maintain wakefulness. Sleep was therefore conceived of as a resting state that the brain enters whenever the input from a number of sensory modalities drops below a certain threshold (Mendelson, 1987, 20).

In the middle of the twentieth century, animal experiments disproved this hypothesis. It was found that when a specific area in the brainstem of a sleeping animal is stimulated with an electrode, diffuse EEG desynchronization and behavioral arousal ensues; and that a slow synchronized EEG coma occurs when the same area is destroyed. Sensory stimulation does not lead to a reversal of this effect, even when the main sensory pathways that connect the periphery to the cortex are preserved (Moruzzi and Magoun, 1949, 471; Hassler, 1971, 27–28; Plum and Posner, 1980, 12). The conclusion drawn from these observations was that the onset of sleep following damage to the brainstem was not, as previously thought, the result of deafferentation, that is, of the interruption of sensory conduction to the brain (Moruzzi and Magoun, 1949, 470); rather, it was now interpreted as the physiological expression of the elimination of a yet undiscovered structure that exerts a waking influence on the cerebrum—of a “subcortical pacemaker diffusely affecting the cortex but lying outside of it” (Plum and Posner, 1980, 12). In 1949, neurophysiologists Moruzzi and Magoun eventually discovered the structure in question: the ascending reticular activating system.^4^

Of the two dimensions of consciousness that we have identified—wakefulness and awareness—the ARAS only regulates the former:

The ARAS influences cortical activity to produce alertness, or cyclical wakefulness, including sleep. Awareness, on the other hand, is principally a product of the thalamus and cerebral cortex, although a functioning ARAS is necessary for it to manifest. (Horne, 2009, 12.2)

What the animal studies showed is also true of human physiology. Put simply, the ARAS functions like a controller for consciousness. When its activity level is high, neural oscillations in the cortex desynchronize and the subject awakes; when the neurons of the ARAS reduce their firing rate, cortical oscillations synchronize again, and the subject becomes drowsy until he or she finally falls asleep.

Arising from the reticular formation of the lower midbrain and upper pons, the ARAS connects to the cortex via three different pathways (Jang and Kwon, 2015, 200, 202). Structural lesions in these strategically important brainstem areas need not exceed the size of a sugar cube to render the ARAS permanently dysfunctional and to induce catastrophic global changes in the electrophysiological activity of the cerebral hemispheres. Given that being awake is a prerequisite of being aware, affected patients become irreversibly comatose (Hassler et al., 1969, 309; Hassler, 1971, 29, 33; Koch, 2010, 20).^5^

This is the configuration in which we are interested for the purpose of this paper. Its relevance as a counter-argument to Lockean accounts of personal identity lies in the fact that tissue damage extensive enough to disable the ARAS is strictly confined to a brain area of which we have reason to assume that it does not house^6^ the neural correlates of long-term memories. Consequently, the persistence of the respective mental states, and thus psychological continuity in the Lockean sense, is *structurally independent* of the persistence of the capacity for wakefulness. From this, it follows that individuals whose ARAS is destroyed and who are therefore never going to regain consciousness nonetheless continue to fulfill the criteria of diachronic existence that Lockean and neo-Lockean accounts endorse.

Let me explain this in greater detail. If the anatomical loci of the ARAS and the brain areas that contain the neural correlates of our memories were entirely congruent, a functional dissociation of arousal and the storage of mental states could not occur. Damage to the respective brain regions would always result in the loss or modification of both the capacity for wakefulness *and* the person’s beliefs, desires, intentions, and so forth. But this is not the case. I have already mentioned two medical conditions that give an indication of this separation: Alzheimer’s disease and amnesia. Alzheimer’s has a profound effect on a subject’s memory, but at least in its earlier stages the disease is not accompanied by a reduction in arousal (Plum and Posner, 1980, 5). The same is true of retrograde amnesia. Amnesic individuals are unable to recall details from their previous life, yet their wakefulness level remains entirely unaffected and consciousness itself is not diminished. This configuration is possible owing to the fact that memories reside predominantly in higher brain regions, whereas large parts of the ARAS—most notably the reticular formation—are located in the phylogenetically older brainstem (Plum and Posner, 1980, 13; Crossman and Neary, 2015, 166–167).^7^

While we are here concerned with the opposite permutation, namely, the persistence of mental states in the absence of the capacity for arousal, it is the same functional segregation that accounts for both scenarios. When the wakefulness component of consciousness is rendered dysfunctional as a result of lesions in the reticular formation or its projections, the neural correlates of memories, and thus of psychological continuity in the Lockean sense, remain unaffected. However, given that being awake is a prerequisite of being aware, the respective mental states cannot become conscious any longer, since “without arousal mediated by the ARAS, awareness is not possible. Even if cortical awareness networks are intact, they remain quiescent without activation by the ARAS” (Edlow et al., 2013, 506).^8^ Beliefs, intentions, and other states thus continue to exist—but they will forever be beyond reach.

In practice, this situation can occur only when the lesion is not extensive enough to stop the brainstem from controlling the organism’s vital homeostatic functions, so that the cerebrum is being kept oxygenated and supplied with glucose and several other substances (Meier, 2022a, 491).^9^ For, if perfusion of the cerebral hemispheres stops and anoxia persists for longer than approximately 3 min, irreparable ischaemic damage to the tissues begins to ensue (Silbernagl and Despopoulos, 2009, 130). In the course of this process, the neural correlates of all memories encoded in the delicate synaptic circuitry are extinguished and psychological continuity is disrupted, in which case Lockeans need no longer regard the comatose entity in the hospital bed as numerically identical with the original person.

Lesions confined to the reticular formation or its projections do indeed occur, their most common causes being ischaemia, hemorrhage, direct trauma, and tumors. Edlow et al. (2013, 506) describe the case of a 62-year-old woman who was admitted to hospital after having suffered severe traumatic brain injury. Spontaneous movement in the extremities was absent, but brainstem-mediated reflexes remained intact. A connectivity analysis revealed that only the subcortical fiber tracts of the ARAS were completely transected, which strongly implicated the latter as the defining substrate of coma (Edlow et al., 2013, 511, 516). Other case studies have confirmed the hypothesis that coma can result exclusively from compromised ARAS structures (Parvizi and Damasio, 2003, 1530; Koch, 2010, 20; Jang and Kim, 2015, 669).

If these clinical reports are correct, and if it is true that the neural correlates of the mental states that constitute psychological continuity do not extend to the brainstem area that is the core of the ARAS, then it follows that the fact that wakefulness cannot be regained owing to a dysfunctional ARAS does not entail that these neural correlates themselves were erased. They are preserved as long as the corresponding brain areas are being oxygenated.

Those who, following Locke, advocate psychological continuity as the criterion of a person’s diachronic existence must consequently conclude that their condition is still fulfilled. For *x* at *t*_1_ (prior to the destruction of the ARAS) and *y* at *t*_2_ (subsequent to the destruction of the ARAS) possess the same number of identical mental states and are therefore sufficiently psychologically connected. The person can *per definitionem* not have ceased to exist when the sole relation that Lockeans endow with significance is not compromised. Hence, the selective destruction of the ARAS is a case of survival according to Lockean accounts of personal identity. For a psychological view, this is a highly unattractive result, given that consciousness can never again materialize in the affected brain.^10^

## IV. FIRST OBJECTION: QUANTITATIVE DEPENDENCE OF MENTAL STATES ON CORTICAL ACTIVATION

Now I consider three objections that proponents of Lockean accounts may raise to the foregoing argument. An important premise is that the persistence of mental states is quantitatively and qualitatively unaffected by the status of the ARAS, for if it were not, there would be no reason to suppose that psychological continuity still obtains when the ARAS is dysfunctional. By *quantitative* independence, I mean that the entities that persist in the brain after the destruction of the ARAS are indeed still the *kinds* of things that Lockeans recognize as constitutive of personal identity across time. *Qualitative* independence denotes the fact that an ARAS defect does not alter the *content* of these states.

Let us begin by examining the quantitative part of the objection. Is a permanently unconscious neural correlate of a mental state still the correlate of a mental state? Or, put differently: is the ARAS an integral component of a mental state’s realizing base? And are Lockeans obliged to accept these unconscious entities as the constitutive elements of psychological continuity?

The essential feature of mental states, it seems, is that they are conscious. *Prima facie*, it therefore appears absurd to suppose that they could persist without this defining attribute. However, even when we are fully awake only a tiny fraction of our mental states is brought to consciousness, while the great majority of them remains unconscious. During episodes of dreamless sleep, coma, and general anesthesia, *none* of the mental states that we possess—or rather their neural correlates—figure in a conscious process.

In the philosophy of mind, authors therefore traditionally distinguish between occurrent and standing mental states (Broad, 1945, 135; Goldman, 1970, 86–87; Block and Fodor, 1972, 168; Shoemaker, 1997, 295; Wollheim, 1999, 1–2; Braddon-Mitchell and Jackson, 2007, 303; Gertler, 2007; Farkas, 2008, 40; Buckwalter, Rose, and Turri, 2015, 753). *Occurrent* (or *transient*) states are those that are being entertained, that is, those that figure in the subject’s mental processes at a given moment in time—like a sudden sensation of cold or an overwhelming feeling of joy. These states are active (Bartlett, 2018a, 11–12). *Standing* (or *dispositional*) states are those that are not currently part of the stream of consciousness. A standing mental state, stored in an individual’s long-term memory, only becomes occurrent when it is integrated into a mental process. One may, for instance, have the standing belief that the moon revolves around the earth, but only when one looks out of the window during a starry night, this belief becomes consciously endorsed and thereby occurrent.^11^

Standing mental states can therefore be conceived of as dispositions to have certain occurrent states (Goldman, 1970, 86; Mele, 2003, 31); or, in other words, (some) occurrent mental states are manifestations of standing states (Bartlett, 2018b, 2). Conversely, occurrent mental states can initiate the formation of standing dispositions when they are laid down in memory: a close encounter with a large spider, for example, may lead to long-lasting arachnophobia in some people (Price, 1969, 244; Wollheim, 1999, 2).^12^

When a subject is dreamlessly sleeping or comatose, he or she possesses, *ipso facto*, only standing mental states. Some states that were occurrent before the stream of consciousness ended are now retained in their dispositional form, while others are simply lost (Buckwalter, Rose, and Turri, 2015, 753). None, however, continue to be occurrent, given that wakefulness is globally absent. Since Lockean accounts of personal identity endeavor to formulate the diachronic persistence conditions of persons for intervals longer than a single day, that is, exceeding periods of awareness uninterrupted by sleep, it follows that standing, rather than occurrent, mental states must form the basis of the relation of psychological continuity that they put forward as the criterion of a person’s persistence.

Although usually not expressed in the above terminology, this is indeed the neo-Lockean position. Sydney Shoemaker, for example, maintains that mental states “need not be conscious; most of them will exist in the way my beliefs about Argentina exist when I am giving no thought to Argentina, or in the way my memories of my schooldays exist when I am sound asleep” (Shoemaker and Swinburne, 1984, 96). To say of a sleeping subject that he retains most of the beliefs, wishes, and personality traits that he has during his waking hours is also in accord with most people’s intuition (Price, 1969, 244; Mele, 2003, 30). As John Searle submits, we understand the notion of an unconscious mental state

only as a possible content of consciousness, only as the sort of thing that, though not conscious, and perhaps impossible to bring to consciousness for various reasons, nonetheless is the *sort of thing* that could be or could have been conscious. (1992, 156)

Thus, unless Lockeans want to stipulate that persons momentarily cease to exist during episodes of dreamless sleep or coma, these unconscious neural correlates of standing mental states are the kinds of entities whose integrity they must accept as sufficient condition of psychological continuity whenever the individual is not awake. Since the function of the ARAS is only to raise the global wakefulness level, it is not a necessary component of these states’ physical realization base; only *occurrent* states require that it be intact. The microstructural configurations in our brain tissues that underlie *standing* mental states are carried forward unimpaired through periods of absent wakefulness. Psychological continuity in the Lockean sense must therefore indeed be quantitatively independent of cortical activation and thereby of a concomitantly operating ARAS. Hence, the objection seems to be unsubstantiated.

Sleep, anesthesia, and transient coma, however, are temporary phenomena. That Lockeans regard standing mental states as constitutive of psychological continuity, they may retort, is really only with the proviso that the former are going to be integrated into a stream of consciousness at a later point in time—that they will become occurrent in the future. If consciousness is ultimately not regained, they may assert, the person has already ceased to exist. Consequently, the ARAS must remain intact. Would this be a convincing reply? Consider the following situation.

Hypnos and Thanatos are brothers who share a genetic predisposition to cardiovascular disease. One evening they go to bed at midnight. Hypnos immediately falls into a peaceful dreamless sleep. At the same time, Thanatos, while still awake, suffers a heart attack and dies quickly. Hypnos continues to sleep until dawn, by which time his heart fails, too. He does not wake up, and eventually his breathing stops.

Did Hypnos and Thanatos cease to exist at the same time? The brothers experienced their last conscious moments at midnight—Thanatos because this was when his circulation collapsed, and Hypnos because, at that time, he fell into a dreamless sleep from which he did not awake.^13^ Although from Hypnos’s perspective it must appear as if he had died at midnight, too, it seems reasonable to assume that the psychological subject Hypnos did not stop existing before dawn. A sleeping person who does not dream has not gone out of existence, irrespective of whether or not consciousness is later regained; for what happens to someone at the end of the night does not retroactively determine his status at any prior point in time. Although Hypnos slipped into unconsciousness at the same time at which Thanatos’s biological life ended, the brothers did not cease to exist simultaneously, that is, with their last conscious moment at midnight. Rather, Hypnos’s psychological continuity extended beyond midnight since the unique structuring of his brain tissues, and thus the neural correlates of his standing mental states, remained intact until, at dawn, his cerebral blood flow broke down and autolysis began to dissolve his hemispheres, thereby irrevocably disrupting psychological continuity. Although Hypnos did ultimately not regain consciousness, there must have been a person present until dawn.

Sleep and coma are characterized by very low or even completely absent ARAS activity. If after midnight Hypnos had suffered a small ischemic brainstem stroke that had caused localized damage to the reticular formation, thereby destroying the core of his ARAS, the ontological status of the neural correlates of his mental states would not have changed in relation to their status during dreamless sleep. Searle (1992, 160) observes that

the possibility of interference by various forms of pathology does not alter the fact that any unconscious intentional state is the sort of thing that is in principle accessible to consciousness. It may be unconscious not only in the sense that it does not *happen* to be conscious then and there, but also in the sense that for one reason or another the agent simply *could not* bring it to consciousness.

Mental states are not only inaccessible when the ARAS is *destroyed*, but whenever it interrupts the stream of consciousness by lowering the level of wakefulness. If one regards the dispositional form that mental states then assume as sufficient for ensuring a person’s persistence during dreamless sleep and transient coma, as Lockeans undoubtedly do, one must *ex hypothesi* also arrive at the same conclusion when the ARAS is defective.

To summarize, Lockeans can account for transient periods of absent consciousness only by postulating that mental states persist independently of cortical activation, that is, in their standing form as mere configurations of oxygenated brain tissue that retains a particular microstructuring. The case of Hypnos and Thanatos shows that this even holds when consciousness is in fact *never* regained. Psychological continuity therefore obtains not only irrespective of whether the neural correlates of mental states are currently incorporated into a conscious process, but also regardless of whether they will *ever again* become so incorporated and become occurrent.

Under ordinary circumstances, this does not present any problem for Lockean accounts, since situations of the latter kind are normally either at least potentially reversible (Hypnos, for instance, did not awake, but he could have), or the defect that keeps the brain from generating consciousness simultaneously also extinguishes the neural correlates of the mental states themselves (as in Thanatos’s case). The sole deviation from this principle occurs when the ARAS is damaged while the rest of the brain remains intact.

Without the potential ever to awake, there is clearly no person present. The neural correlates of psychological continuity in a brain that has become incapable of reaching an adequate level of wakefulness no more constitute a living person than an indecipherable blueprint for a house constitutes a building. Consequently, if advocates of memory-based accounts adhere to their criterion of personal identity, they are forced either to hold that the existence of persons ends when they fall into a dreamless sleep, are anesthetized, or enter a transient coma (and to assume that numerically different subjects come into being upon awakening) or to classify irreversibly comatose individuals as Lockean persons. Neither option seems tolerable.

## V. SECOND OBJECTION: QUALITATIVE DEPENDENCE OF MENTAL STATES ON CORTICAL ACTIVATION

Lockeans may accept that a defect of the ARAS does not extinguish the neural correlates of standing mental states, but argue instead that it changes their respective content. This is the *qualitative* objection. Since, according to these views, transtemporal psychological connections are established only between mental states that are qualitatively identical between *t*_1_ and *t*_2_, such as the same belief or the same intention, any substantial alteration of these states’ content would disrupt psychological continuity, in which case the Lockean condition of diachronic persistence would cease to be fulfilled. The Lockean person would then indeed no longer exist and this paper’s argument against this view of personal identity would be unfounded.

Psychologist Hans Eysenck famously suggested that dissimilarities in cortical arousal are responsible for behavioral differences between introverts and extraverts. He hypothesized that the latter exhibit lower arousal levels, which prompt them to seek a greater amount of external stimulation than introverts (Eysenck, 2006). Arousal is a function of the ARAS. Hence, if Eysenck was right, the envisaged situation in which the neural correlates of standing mental states persist unaltered while consciousness is irreversibly absent could not occur—for a comatose individual’s mental states would not only be withdrawn from any conscious process but, devoid of the influence of the ARAS, actually be *different*. Does Eysenck’s theory therefore refute our argument?

As noted in the beginning, Locke only regarded recollections of autobiographical events as the basis of psychological connectedness. Personality traits, with which Eysenck was concerned, do not fall into this category. Since arousal levels cannot interfere with autobiographical memories in the way that Eysenck took them to impact on personality traits, Locke himself could not employ any dependence that might exist between ARAS activity and temperament against our argument.

As also previously mentioned, however, neo-Lockean views of personal identity endeavor to correct the imbalance towards autobiographical recollection by recognizing many different classes of mental states as the basis of psychological continuity. Since personality traits are among these recognized types, there would, if Eysenck’s theory were correct, indeed be one class of mental states that is constitutive of a person’s persistence while not being qualitatively independent of the prevalent degree of arousal.

However, there is no indication that the level of wakefulness modifies any other class of standing mental states: a person’s *belief* that London is the capital of the United Kingdom persists unchanged regardless of whether he is drowsy or highly alert, his *memory* of seeing the Tower Bridge remains stable through periods of sleepiness, and his *wish* to meet the Queen does not change during general anesthesia. Whether these states can be *accessed*, that is, whether they can become conscious or play a subconscious role, does depend on whether wakefulness is being generated by the ARAS. A comatose individual cannot put to use his beliefs or intentions, just as he cannot relive his memories. Qualitatively, however, the mental states remain unaltered. It is this very dichotomy on which our argument against the Lockean views rests. We can therefore conclude that since personality traits constitute only one of the many types of mental states that neo-Lockean approaches recognize, Eysenck’s theory—which remains controversial even among psychologists^14^—does not present a successful objection.

## VI. THIRD OBJECTION: REVERSIBILITY OF ARAS DEFECTS

“Irreversible” is a polysemous term. There are different types of irreversibility, and it may be decisive to which a dysfunctional ARAS belongs. The impossibility of restoring wakefulness in a certain brain may be logical, nomological, metaphysical, or merely technical (Meier, 2022b, 221). If an ARAS defect should fall into the last of these categories, one could argue that Lockean persons would survive this condition since the standing mental states, which remain physically realized, could *in principle* still become conscious—if the appropriate technology were available. There would, at a future time, be an event that would count as the arousal of numerically the same person who is related through psychological continuity to the one who once suffered the destruction of his or her ARAS. The subject’s mental states would in this case only be de facto inaccessible, that is, in relation to the technology available today.^15^ Since death is *per definitionem* an irreversible state, the person would have to remain in existence as long as there is the possibility for him or her to regain consciousness at any subsequent point in time.^16^ Do we have reason to regard the ARAS as a potentially replaceable structure?

Two considerations, it appears, are relevant in this regard. The first, which one may term the *technological* problem, is the question as to whether we can expect to develop a functional prosthesis. The second, which one could call the *metaphysical* problem, is the question as to whether someone who awakes with the help of this implanted device would still count as numerically the same psychological individual. If the answer to this latter question is negative, then the subject can *in principle* not return to consciousness, even if one answers the first question in the affirmative, that is, even if a replacement is *in practice* technologically feasible.

Both problems are extremely hard to resolve, and I am not pretending to have answers to them. The precariousness of the technological problem stems from the fact that long-term scientific progress is largely unpredictable.^17^ The metaphysical problem, on the other hand, is vexed because we have not yet understood how nervous tissue brings forth mental phenomena. Whether the realization of a person’s consciousness is conditional on its supervening on the same brainstem substrate for generating wakefulness is something we do not know—just as we do not know this in the case of awareness and the cerebrum.^18^ The best we can currently do is, first, carefully to consider the relevant physiological facts and, second, to take into consideration the relatively scarce clinical case reports that we have. This is what I am going to do next.

The ARAS is often portrayed as an on/off-switch for consciousness, which gives the impression that it is a relatively primitive structure. As we have already determined, however, wakefulness is not a binary affair but comes in many degrees. The ARAS should therefore rather be conceived of as a dimmer (Damasio, 2010, 159). It is a complex network that originates from multiple brainstem source nuclei, projects to the cortex via thalamic and extrathalamic pathways, and releases various types of neurotransmitters (Parvizi and Damasio, 2003, 1525; Zeman, 2006, 363; Edlow et al., 2012, 531; Jang and Kwon, 2015, 200–201). The ARAS does not simply monodirectionally stimulate the cortex, but is itself influenced by the hypothalamus and the basal forebrain that reciprocally innervate the reticular formation, thereby providing a feedback mechanism that modulates ARAS activity (Plum and Posner, 1980, 13–14; Moll et al., 2009, 126, 140). Some authors even suspect the existence of several ascending activating systems working in parallel (Robbins, 1997; Machado, 1999, 157) and argue that the role of brainstem structures in the generation of consciousness has been underestimated (Merker, 2007).

How successful have doctors been at treating defects in this complex structure? In the late 1960s, Rolf Hassler et al. (1969, 306) and his team tried to restore consciousness in an unresponsive patient by inserting electrodes into the basal part of the right pallidum and the left latero-polar nucleus of the thalamus. In reaction to this stimulation, the patient opened his eyes and exhibited spontaneous movements. Unintelligible vocalization occurred and “the level of consciousness was definitely improved” (Hassler et al., 1969, 308–309). More recent studies have largely confirmed both the therapeutic potential of electrical ARAS stimulation as well as the limitations of this method (Sturm et al., 1979; Cohadon and Richer, 1993; Yamamoto and Katayama, 2005; Schiff et al., 2007; Moll et al., 2009; Koch, 2010). Is the destruction of the wakefulness component of consciousness becoming reversible?

Neither Hassler’s group nor those who conducted the subsequent studies in fact substituted an electrical circuit for a defective ARAS. There is a big difference between stimulating a certain brain area and actually *replacing* its function. In the former case, electrical pulses interfere with neural networks at the target site to produce a desired outcome. This necessitates that the respective structures be largely intact. In the latter case, however, the entire structure would have to be exchanged for a substitute. What Hassler et al. achieved was restoring the function of an only partly compromised ARAS from a lower to a somewhat higher level of arousal:

The *anatomically undamaged* neurones or parts of this non-specific system should be induced to take up again their spontaneous ascending activation of the cortex, necessary for EEG arousal and awareness, by long-term stimulation. (1969, 309)^19^

Although a remarkable accomplishment, especially in view of the fact that the study was conducted as early as in the 1960s, interventions of this kind are still very far from actually substituting for a dysfunctional ARAS, and not much progress has been made since. In conclusion, it is at present at least technologically impossible to replace or otherwise restore the function of an entirely destroyed activating system. Whether this also presents a metaphysical impossibility, for instance, because the very arousal network that developed along with the cerebrum is needed for a particular individual to regain consciousness, we cannot say. The high complexity of the ARAS could be an indication that this may well be so.

## VII. CONCLUSION

Proponents of Lockean and neo-Lockean accounts of personal identity maintain that persons persist diachronically by virtue of being psychologically continuous with their former selves. Other authors pointed out that memory-affecting disorders like Alzheimer’s disease and amnesia pose threats to these views as demented and amnesic individuals are still conscious while psychological continuity is disrupted. I have been investigating whether the opposite permutation also exists: can one retain psychological continuity in the permanent absence of the capacity for consciousness? And if so, what are the consequences for Lockean accounts?

The persistence of the neural correlates of a person’s standing mental states, and consequently of psychological continuity, is conditional on the cerebrum being oxygenated and supplied with glucose. Conversely, the retention of the capacity for consciousness is dependent on the integrity of *two* anatomically distinct loci: the cerebrum, which contributes the awareness component to consciousness, and the ARAS, which is responsible for wakefulness and originates in the brainstem. Since wakefulness is a precondition of awareness, the destruction of the ARAS alone results in the permanent loss of consciousness. However, due to the fact that the neural correlates of the mental states that constitute an individual’s long-term memory do not overlap with the core area of the ARAS, psychological continuity remains unaffected by damage to the latter. The Lockean condition of a person’s diachronic existence is therefore still fulfilled—although it is obvious that, devoid of the structural prerequisites of wakefulness, the subject must have ceased to exist.

I considered three objections that Lockeans might raise. They might insist that the ARAS is a constitutive part of the realization base of a mental state, so that whatever physical traces remain in the oxygenated brain tissues after the irreversible loss of wakefulness should not be recognized as proper neural correlates of mental states. Psychological continuity would be disrupted and the Lockean criterion of diachronic persistence would no longer apply. I replied that if everyday phenomena like dreamless sleep and transient coma are not to present unsurmountable obstacles to their theory, Lockeans must regard as sufficient the possession of standing mental states and cannot demand that occurrent states be retained, too. The former, however, persist independently of cortical activation and thus of ARAS activity. I introduced a thought experiment to show that this is even the case when consciousness is in fact never regained. As dreamless sleep, transient coma, and permanent ARAS defects are therefore on a par in all relevant respects, Lockeans must recognize the specific unconscious microstructural configurations of cerebral tissue as proper correlates of standing mental states either in all three cases or in none of them.

Lockeans may accept that a lesion in the ARAS does not extinguish the neural correlates of mental states, but instead maintain that it changes the states’ content. As psychological continuity only obtains between qualitatively identical mental states, this relation would then be disrupted. According to Eysenck’s influential model of personality, dissimilarities in cortical arousal correlate with behavioral differences, which, if true, would mean that certain character traits are as much a consequence of ARAS activity as they are of microstructural dispositions in the cerebrum. Against this objection I contended that, even if Eysenck’s model were physiologically accurate, the charge would only pertain to a subcategory of the many types of mental states that neo-Lockeans generally acknowledge. In all other cases, the influence that the ARAS exerts on mental states is confined to a global regulation of their accessibility to consciousness by generating wakefulness.

Lastly, Lockeans could assert that a coma resulting from the destruction of the ARAS is not an irreversible condition, since the relevant brain structures could at a future time be repaired or replaced. I put forward a technical and a metaphysical objection to this supposition. The ARAS is a highly complex network that provides widespread innervation to various areas of the cerebrum. Clinical studies showed some success in stimulating a partly defective ARAS, but only when the principal neural structures were largely intact. That functional prostheses will ever be available seems therefore improbable. Moreover, it is metaphysically unclear whether an individual who awoke with such a prosthesis would still be the same numerical subject, given that one of the two elements of the capacity for consciousness would have been radically modified. It may therefore be reasonable to assume that, following the destruction of the ARAS, the capacity for consciousness is not contingently but necessarily absent.

In their current form, Lockean accounts of personal identity have untenable consequences when confronted with these neurophysiological facts. When there are real-life cases in which the Lockean condition for diachronic existence is fulfilled in spite of the irreversible absence of any conscious mental activity, all motivation for holding on to what purports to be a *psychological* view vanishes. In order to account for the neurophysiological peculiarities of the human brain and to avoid the absurd implication that psychological persons continue to exist even when they neither can, nor ever will, awake or even dream, an *accessibility clause* would have to be added to the condition for diachronic persistence. The definition would then read:

An individual *x* at *t*_1_is sufficiently psychologically continuous, and hence identical, with an individual *y* at *t*_2_ if and only if there are a certain number of psychological connections that form overlapping chains between those two points in time, *and the mental states that are the basis of these connections remain accessible to consciousness.*

Alternative formulations of this addendum would specify that the *capacity for wakefulness must be preserved* or that *any interruption in the stream of consciousness between t*_*1*_*and t*_*2*_*must be reversible*.^20^ The gist is that the brain in whose substrate the neural correlates of the person’s mental states are microstructurally encoded has not irreversibly lost its ability to generate wakefulness. Amendments to this effect can, I believe, rescue Lockean accounts from the challenge that this article poses. Irrespective of whether the Lockean conception of our diachronic persistence is convincing in the end—the important function that the ARAS fulfills in our mental lives should figure in the criteria of any psychological account of personal identity.

## Footnotes

1 By “consciousness” Locke meant “memory,” as Thomas Reid (1878, III.III, § 2) noted. See also Noonan (2003, 43–44).

2 There are two types of neo-Lockeanism: the constitutionalist (Johnston, 1992; Baker, 2000, 2007) and the non-constitutionalist (Shoemaker, 1970, 1999, 2008; Parfit, 1971, 1987; Perry, 1972; Lewis, 1976; Shoemaker and Swinburne, 1984). My argument primarily addresses the latter kind.

3 How many connections are required for psychological continuity to be strong enough is a moot point. Parfit (1987, 206) suggested that half the number of direct connections that hold over any day in the life of an ordinary individual should be regarded as sufficient.

4 See also Dempsey and Morison (1941) and Mauthner (1890).

5 Even in REM sleep, in which the subject is aware of the contents of his dreams while not being fully conscious, a certain degree of wakefulness, and thus of cortical activation, is required for these dreams to manifest (Evans, 2003, 2–3; Laureys, 2005, 556; Windt, Nielsen, and Thompson, 2016).

6 This is to be understood in the most neutral sense possible. I withhold judgment on whether identity theory, double-aspect theory, property dualism, functionalism, or any other of the theories that have been proposed accurately describes the relation between mental properties and their neural substrate. Especially in the phenomenological tradition, the notion of the brain “containing” any information is often rejected (Fuchs, 2017, 58–64). Since Lockeans generally do not dispute this assumption, however, I do not question it here.

7 An interesting and possibly relevant difference that I cannot here pursue is the divergence in susceptibility to memory-affecting diseases between episodic, semantic, and procedural memory. Retrograde amnesia, for instance, usually spares procedural memories (Squire and Kandel, 1999, 14; Machado et al., 2009, 334).

8 See also Jang and Kim (2015, 671) and Parvizi and Damasio (2003, 1531–32).

9 A small number of cases have been reported in which the cerebrum continued to be perfused, even though the brainstem was nearly entirely destroyed (Deliyannakis, Ioannou, and Davaroukas, 1975; Kosteljanetz et al., 1988; Schlake and Roosen, 2001, 69–70; Zwarts, Kornips, and Vogels, 2001).

10 Views that focus on the continuity of the capacity for consciousness per se rather than on the preservation of long-term memories, like Jeff McMahan’s (2002, 66–94) Embodied Mind Account and Peter Unger’s (1990) Core Psychology Approach, are not afflicted with this problem. While these accounts belong to the large family of psychological views of personal identity, they are not of the Lockean type since they explicitly deny that standing mental states play a role in the diachronic persistence of persons. Consequently, they are beyond the scope of this paper. I analyze these views in Meier (2020).

11 Standing mental states must not be confused with what is commonly referred to as *subconscious* states. Subconscious states are emotions, wishes, and so forth, of which the subject is also not aware, but which are nonetheless active and may therefore still exert an influence on his actions or on his other mental states (Mele, 2003, 30–31; Farkas, 2008, 40–41, 45–48; Freud, 2016, 7; Bartlett, 2018a, 7–8). When, for example, a repressed feeling of guilt guides someone’s behavior, the subject may not be aware of its existence because it operates below the level of conscious reflection.

12 The exact nature of the relation between these two basic modes of mental states has been the subject of a long debate in which I cannot here engage. See, for example, Bartlett (2018a); Farkas (2008, 40–45), Gertler (2007), Crane (2001, 105–8), Wollheim (1999, 2–3), and Block and Fodor (1972, 168–69).

13 Should it be objected that even in a dreamless sleep the cerebrum is not totally idle, one could exchange this scenario for pharmacologically or hypothermically induced burst suppression followed by a lethal injection.

14 See, for example, Gray’s (1981) classic critique and Hagemann and Naumann’s (2009) more recent study.

15 I owe the idea of applying this distinction to the question at hand to Jeff McMahan (1995, 105). Besides repairing or replacing the ARAS, a further, highly hypothetical way of accessing the mental states might be considered by some, namely, extracting the latter from the cerebrum and feeding them into an emulation. In *Whole Brain Emulation: A Roadmap*, Sandberg and Bostrom (2008, 83) paint a very optimistic picture of the technological feasibility of such a project. As a thought experiment, Bernard Williams (1970, 162–63) imagined a similar situation. The assumption that a mental state is an entity that could simply be detached from its neural carrier substance and, like a digital file, be transferred to different media, is extremely speculative. I therefore do not pursue this idea. For a critique of thought experimentation as a method for deciding questions in personal identity, see Meier (2022b).

16 Under *tertium non datur* conditions, that is. Two alternative options, which I am not considering, are postulating a third, “suspended” state between existence and non-existence, and deeming metaphysically sound what Locke (2008, II.XXVII, § 1) explicitly denied, namely, that one and the same thing can have two beginnings.

17 To give just two examples of world-leading experts in their fields whose predictions turned out to be utterly misguided: no less a person than Albert Einstein remarked that “there is not the slightest indication” that nuclear energy “will ever be obtainable” (Pittsburgh Post-Gazette, 1934). And when Thomas Edison was asked what technological progress the coming hundred years would bring, he answered that iron could soon be turned into gold since scientists were “already on the verge of discovering the secret of transmuting metals” (The Miami Metropolis, 1911).

18 See also Unger’s (1990, 143–45) case of the “taped brainstem.” His conclusions are, however, marred by a conflation of the presence and absence of wakefulness with the distinction between conscious and unconscious information processing in the brain. For the latter, see Block (1995). See also McMahan’s (2002, 442–443; 1995, 104–108) excellent discussions of the relevance of the ARAS to his own account of personal identity.

19 Emphasis added. Due to the redundancy of connections from the ARAS brainstem nuclei to higher brain regions, the ARAS appears to have the capacity to recover when only a certain fraction of its pathways are severed (Edlow et al., 2013, 521).

20 From the hypothetical scenario featuring Hypnos and Thanatos, we learned that the *capacity* for reversibility is sufficient; whether a reactivation of the mental states actually occurs, that is, whether this capacity is indeed realized and consciousness is regained, is indecisive.
